# Supplementary figures and images for: Broad similarities in shoulder muscle architecture and organization across two amniotes: implications for reconstructing non-mammalian synapsids
Source: PeerJ. 2020 Feb 18;8:e8556. doi: 10.7717/peerj.8556 (PMC7034385; doi:10.7717/peerj.8556)

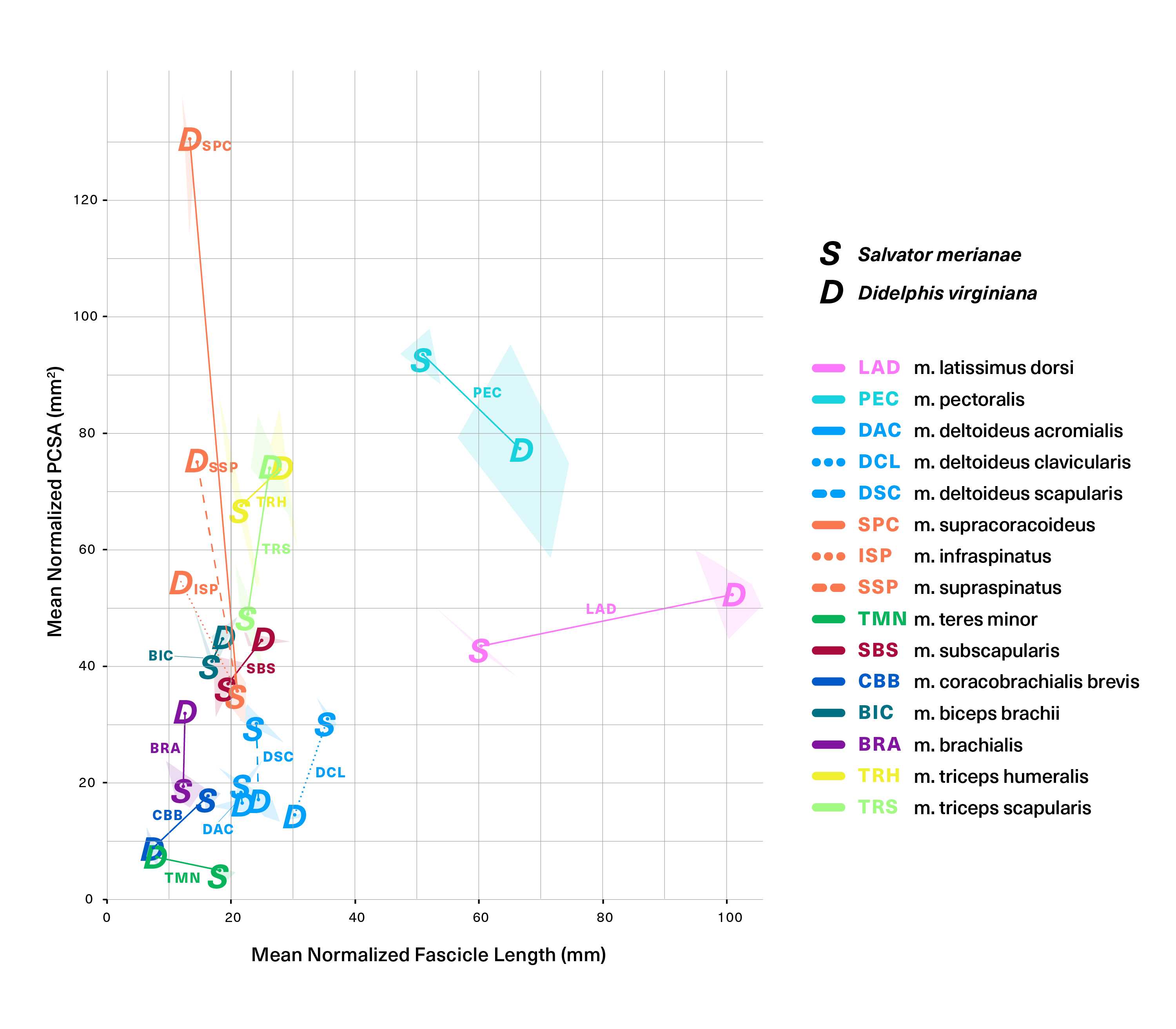

Supplement: Supplemental Information 1 — Functional morphospace comparing normalized PCSA against normalized fascicle length. Muscles tend to vary along either one axis or the other, consistent with a tradeoff between force production (Y-axis) and working range (X-axis). Muscle abbreviations follow Fig. 2. [file peerj-08-8556-s001.png]

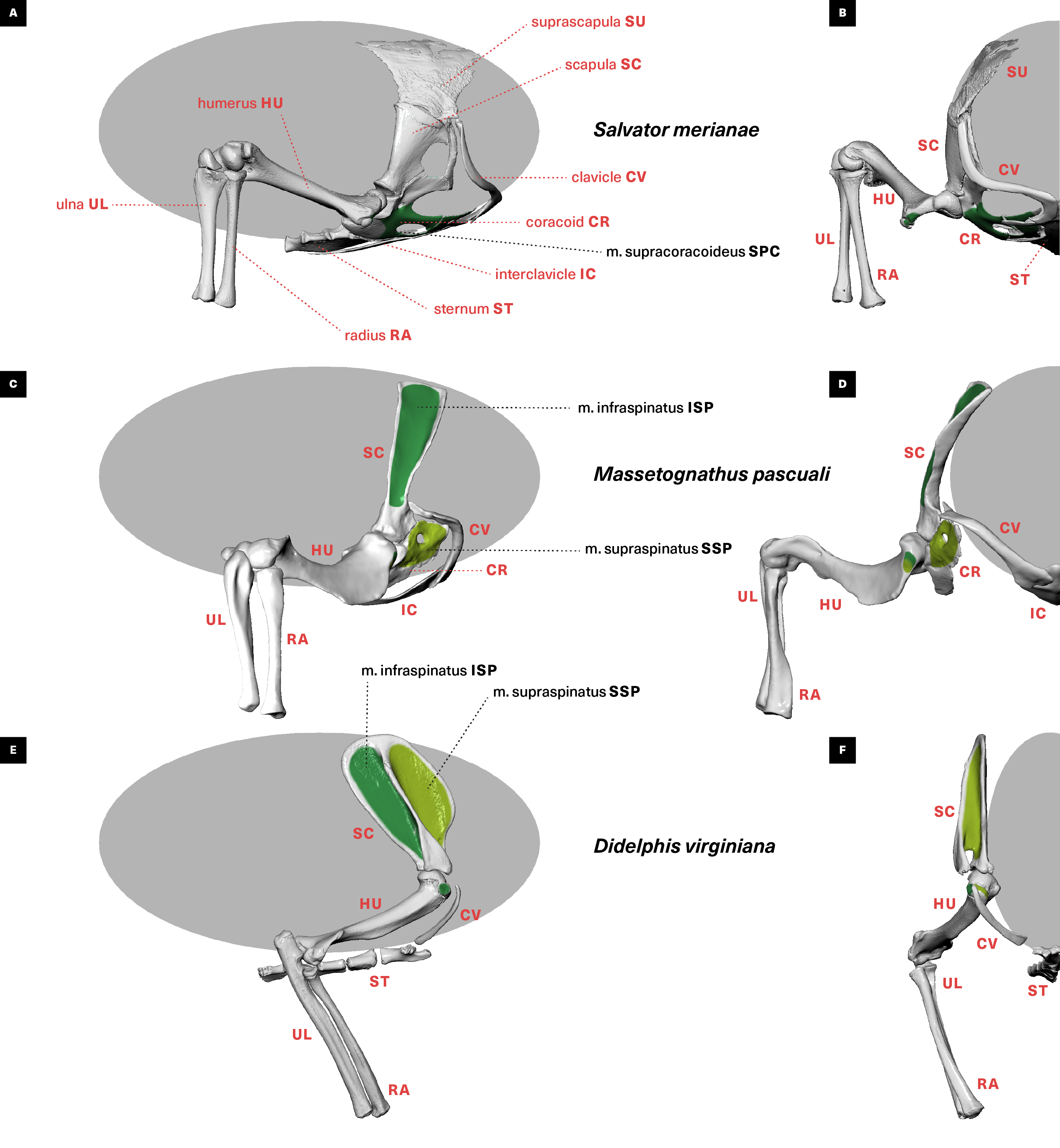

Supplement: Supplemental Information 2 — All animals shown in right lateral view (left) and cranial view (right). M. pascuali is depicted in a hypothetical neutral pose, reflecting the middle of each joint’s range of motion (see Lai, Biewener & Pierce, 2018). S. merianae and D. virginiana poses taken from 3D videoradiography. While in Massetognathus the ventral portion of the scapulocoracoid comprises separate procoracoid and metacoracoid elements (sensu Vickaryous & Hall, 2006), here they are collectively termed the “coracoid” for simplicity. Muscle origin areas for m. supracoracoideus, m. infraspinatus, and m. supraspinatus are shown in dark green (mm. supracoracoideus and infraspinatus) and light green (m. supraspinatus) respectively. [file peerj-08-8556-s002.png]
