# Supplementary material for: Broad similarities in shoulder muscle architecture and organization across two amniotes: implications for reconstructing non-mammalian synapsids
Source: PeerJ. 2020 Feb 18;8:e8556. doi: 10.7717/peerj.8556 (PMC7034385; doi:10.7717/peerj.8556)
Supplement: Supplemental Information 3 — Individuals depicted in Fig. 2 are marked with an asterisk (*). [file peerj-08-8556-s003.docx]

### **Supplemental text S1: Homologies of m. deltoideus acromialis and m. scapulohumeralis anterior**

**m. deltoideus acromialis**

Most workers describe m. deltoideus as comprising two heads (scapular and clavicular) in extant reptiles, and three heads (scapular, clavicular, and acromial) in extant mammals (although the existence of an acromial head in monotremes is disputed (1,2). While dissecting the tegus, we located a muscle in the approximate location described for m. scapulohumeralis anterior, but found overwhelming evidence for its homology with the opossum m. deltoideus acromialis when we examined its nerve supply and topology. We traced this muscle’s innervation and found it to be supplied by a branch of the axillary nerve, adjacent to the branch supplying the clavicular head of the deltoid. Caudal to the glenoid, m. deltoideus acromialis is separated from m. scapulohumeralis anterior by the cranio-dorsal cruciate ligament (3) connecting the scapula to the humerus, adhering to the lateral side of the ligament rather than passing medial to it (Fig. 2). Based on its relationship with the cranio-dorsal ligament, we reject the possibility that this muscle represents a second division of m. scapulohumeralis anterior (as described in some other lizards (4). On the same basis, we also reject the possibility that m. deltoideus acromialis is the true scapulohumeralis anterior, which would imply that m. scapulohumeralis anterior in S*alvator* is actually m. scapulohumeralis posterior, as described in *Sphenodon, Varanus* and *Agama* (4–6)—all available descriptions of scapulohumeralis posterior describe it as passing medial to the cranio-dorsal ligament, rather than lateral*.* We consider the gross morphology, topology, innervation, and architecture of this third deltoid head to be compelling evidence for homology with the mammalian m. deltoideus acromialis, also found in the lizard *Chamaeleo* (7).

**m. scapulohumeralis anterior**

All living sauropsids except turtles possess either a scapulohumeralis anterior (squamates), a scapulohumeralis posterior (crocodilians), or both (birds, *Sphenodon,* and the lizards *Varanus* and *Agama*) (4,6,8), all of which were likely derived from a single scapulohumeralis muscle mass in basal sauropsids (5,9,10). Homology between m. teres minor and m. scapulohumeralis anterior was first hypothesized by Romer (11), and later corroborated by Cheng (12) on the basis of developmental data from the lizard *Lacerta agilis* as well as *Didelphis virginiana*. Both muscles are present relatively early in ontogeny*,* both arise from an anlage shared with the future deltoids, and both are supplied by a branch of the axillary nerve.

One critique of this interpretation cites as evidence the coexistence of both m. teres minor and m. scapulohumeralis anterior in the adult form of extant monotremes, and the supposed innervation of the scapulohumeralis anterior by the radial nerve (13). To the first point, muscular homologies between monotremes and other tetrapods are historically a matter of some controversy (2,14–16), and non-conjunction (i.e., the assertion that two structures cannot be homologues if they coexist in the same organism) is considered a weak test of homology in scenarios involving the transformation of structures (e.g. 17). To illustrate this with a different example, m. supracoracoideus exists alongside its derivatives mm. infraspinatus and supraspinatus in extant monotremes. The non-conjunction principle holds that these muscles cannot be homologues, yet their homology is well established and accepted without controversy (1,2).

Regarding the question of innervation, while Lecuru-Renous (4) state that the scapulohumeralis anterior muscle is innervated by a nerve associated with the radial trunk, their figures show mostly axillary trunk contribution with a small branch from the radial trunk, in common with Romer’s developmental series. Fürbringer corroborates an axillary association for this nerve, describing it as arising from the same trunk as the nerve supplying m. deltoideus clavicularis (5). Our dissections also confirm a consistent axillary innervation for the scapulohumeralis anterior. A final piece of evidence is given by Lecuru-Renous, who finds that the scapulohumeralis anterior nerve often gives rise to a long, cutaneous nerve (4). In humans, the teres minor is innervated by the posterior branch of the axillary nerve, which also gives rise to a long, cutaneous nerve (the superior lateral cutaneous nerve) (18). Given the combined weight of topological, developmental, and neuroanatomical evidence, we follow Romer and Cheng in interpreting m. scapulohumeralis anterior and m. teres minor as homologues.

1. Diogo R, Abdala V. Comparative anatomy, homologies and evolution of the pectoral muscles of bony fish and tetrapods: a new insight. J Morphol. 2007 Jun;268(6):504–17.

2. Gambaryan PP, Kuznetsov AN, Panyutina AA, Gerasimov SV. Shoulder girdle and forelimb myology of extant Monotremata. Russ J Theriol. 2015;14(1):1–56.

3. Haines RW. The shoulder joint of lizards and the primitive reptilian shoulder mechanism. J Anat. 1952 Oct;86(4):412–22.

4. Lecuru-Renous S. Myologie et innervation du membre antérieur des Lacertiliens. Paris: Éditions du Muséum; 1968.

5. Fürbringer M. Zur vergleichenden Anatomie des Brustschulterapparates und der Schultermuskeln. IV Teil. Jena ische Zeitschr Naturwiss, Jena. 1900;34:351.

6. Jenkins FA, Goslow GE. The functional anatomy of the shoulder of the savannah monitor lizard *(Varanus exanthematicus)*. J Morphol. 1983 Feb 1;175(2):195–216.

7. Molnar JL, Diaz RE Jr, Skorka T, Dagliyan G, Diogo R. Comparative musculoskeletal anatomy of chameleon limbs, with implications for the evolution of arboreal locomotion in lizards and for teratology. J Morphol [Internet]. 2017 May 18; Available from: http://dx.doi.org/10.1002/jmor.20708

8. Abdala V, Diogo R. Comparative anatomy, homologies and evolution of the pectoral and forelimb musculature of tetrapods with special attention to extant limbed amphibians and reptiles. J Anat. 2010 Nov;217(5):536–73.

9. Miner RW. The pectoral limb of *Eryops* and other primitive tetrapods. Bulletin of the AMNH. 1925;51(7):145–312.

10. Holmes R. The osteology and musculature of the pectoral limb of small captorhinids. J Morphol. 1977 Apr 1;152(1):101–40.

11. Romer AS. The development of tetrapod limb musculature—the shoulder region of *Lacerta*. J Morphol. 1944;74(1):1–41.

12. Cheng C-C. The development of the shoulder region of the opossum, *Didelphys virginiana,* with special reference to the musculature. J Morphol. 1955 Nov 1;97(3):415–71.

13. Jouffroy FKL, Saban J, Souteyrand-Boulenger R. Mammifères: musculature des membres, musculature peaucière, musculature des monotrèmes. Arthrologie. Paris: Masson; 1971.

14. Howell AB. Morphogenesis of the Shoulder Architecture. Part V. Monotremata. Q Rev Biol. 1937;12(2):191–205.

15. McKay WJS. The morphology of the muscles of the shoulder-girdle in monotremes. Proc Linn Soc N S W. 1894;9:263–360.

16. Walter LR. Appendicular Musculature in the Echidna, *Tachyglossus aculeatus* (Monotremata, Tachyglossidae). Aust J Zool. 1988 Jan 1;36(1):65–81.

17. Patterson C. Morphological Characters and Homology. In: Joysey KA, Friday AE, editors. Problems of Phylogenetic Reconstruction. Academic Press; 1982. p. 21–74.

18. Drake R, Vogl AW, Mitchell AW. Gray’s anatomy for students. Toronto, Ontario, Canada: Elsevier Health Sciences; 2014.
