# Supplementary material for: Broad similarities in shoulder muscle architecture and organization across two amniotes: implications for reconstructing non-mammalian synapsids
Source: PeerJ. 2020 Feb 18;8:e8556. doi: 10.7717/peerj.8556 (PMC7034385; doi:10.7717/peerj.8556)
Supplement: Supplemental Information 4 [file peerj-08-8556-s004.docx]

|  | ***Salvator merianae*** | | | | ***Didelphis virginiana*** | | | |
| --- | --- | --- | --- | --- | --- | --- | --- | --- |
| **Specimen** | SEP 71 | SEP 104 | SEP 105 | SEP 111 | SEP 85 | SEP 88 | SEP 92 | SEP 101 |
| **Body mass (kg)** | 1.40 | 1.31 | 1.18 | 1.41 | 1.56 | 1.06 | 1.21 | 1.57 |
