# Supplementary material for: Broad similarities in shoulder muscle architecture and organization across two amniotes: implications for reconstructing non-mammalian synapsids
Source: PeerJ. 2020 Feb 18;8:e8556. doi: 10.7717/peerj.8556 (PMC7034385; doi:10.7717/peerj.8556)
Supplement: Supplemental Information 5 — P-values shown are adjusted for multiple comparisons using the Benjamini–Hochberg procedure. Muscle abbreviations follow Fig. 2. [file peerj-08-8556-s005.docx]

|  |  |  |  |  |
| --- | --- | --- | --- | --- |
|  | **Mean Pennation Angle (θ)** | Mean Normalized PCSA | Mean Normalized Fascicle Length (L_f_) | Mean Normalized Muscle Mass (M_m_) |
| LAD | N/A | 0.195 | 0.006 | 0.0042 |
| PEC | N/A | 0.18857143 | 0.04 | 0.56727273 |
| DAC | 0.0264 | 0.38181818 | 0.92 | 0.86 |
| DCL | N/A | 0.01992 | 0.03 | 8.00E-04 |
| DSC | 0.91 | 0.0147 | 0.92 | 8.00E-04 |
| SPC | 8.80E-04 | 0.00384 | 0.00126 | 0.002328 |
| SBS | 0.91 | 0.18857143 | 0.07371429 | 0.024 |
| CBB | N/A | 0.0147 | 3.96E-04 | 0.00147 |
| BIC | 0.48 | 0.372 | 0.30666667 | 0.0624 |
| **TRS** | 0.56 | 0.0147 | 0.18 | 9.24E-05 |
| TRH | 0.56 | 0.52 | 0.04 | 0.00735 |
| BRA | 0.248 | 0.2 | 0.92 | 0.00565714 |
