# Supplementary material for: Broad similarities in shoulder muscle architecture and organization across two amniotes: implications for reconstructing non-mammalian synapsids
Source: PeerJ. 2020 Feb 18;8:e8556. doi: 10.7717/peerj.8556 (PMC7034385; doi:10.7717/peerj.8556)
Supplement: Supplemental Information 6 — Following Bates et al. (2015), two separate estimates of uncertainty are given: upper and lower bounds are calculated from the Ordinary Least Squares (OLS) model reported in Campione & Evans (2012), using (1) mean percent prediction error (PPE) and (2) 95% prediction intervals (95% PI). This method accurately predicts the body masses of Salvator and Didelphis: actual measured body masses of both living amniotes are included within both the PPE and the 95% PI bounds. The narrower PPE bounds are used to calculate error in the scaled muscle architecture properties of Massetognathus pascuali (Fig. 9). [file peerj-08-8556-s006.docx]

|  | ***Salvator merianae*** | ***Didelphis virginiana*** | ***Massetognathus pascuali*** |
| --- | --- | --- | --- |
| **Specimen** | SEP 73 | SEP 101 | MCZVP 3691 |
| **Humerus minimum diaphyseal circumference (mm)** | 13.049±0.058 | 16.982±0.225 | 17.825±0.109 |
| **Femur minimum diaphyseal circumference (mm)** | 16.711±0.005 | 18.638±0.067 | 17.672±0.531 |
| **Log stylopodial circumference** | 1.474 | 1.552 | 1.550 |
| **Measured body mass (kg)** | 1.036 | 1.568 | N/A |
| **Predicted body mass (kg)** | 0.885 | 1.451 | 1.437 |
| **Upper and lower bounds**  **based on PPE (kg)** | 1.112-0.658 | 1.823-1.079 | 1.806-1.069 |
| **Upper and lower bounds**  **based on 95% PI (kg)** | 1.636-0.479 | 2.681-0.786 | 2.655-0.778 |
