# Supplementary material for: Broad similarities in shoulder muscle architecture and organization across two amniotes: implications for reconstructing non-mammalian synapsids
Source: PeerJ. 2020 Feb 18;8:e8556. doi: 10.7717/peerj.8556 (PMC7034385; doi:10.7717/peerj.8556)
Supplement: Supplemental Information 7 — In Massetognathus pascuali, the m. infraspinatus (ISP) origin area resembles that of the Didelphis m. infraspinatus, whereas the m. supraspinatus (SSP) origin area resembles that of the Salvator m. supracoracoideus (SPC) (Fig. S2). Thus, the “low” estimate—using the PCSA: muscle origin area index of the Didelphis ISP for the Massetognathus ISP, and the index of the Salvator SPC for the Massetognathus SSP—constitutes a morphologically-informed best guess (marked with an asterisk (*)). An alternative “high” estimate uses Salvator SPC for Massetognathus ISP, and Didelphis SSP for Massetognathus SSP. Muscle origin areas are shown in Fig. S2. [file peerj-08-8556-s007.docx]

| ***Salvator merianae*** | | | |
| --- | --- | --- | --- |
|  | **Normalized**  **PCSA (mm^2^)** | **Normalized muscle**  **origin area (mm^2^)** | **PCSA:muscle**  **origin area index** |
| **SPC** | 35.50 | 197.00 | 0.180 |
| ***Didelphis virginiana*** | | | |
|  | **Normalized**  **PCSA (mm^2^)** | **Normalized muscle**  **origin area (mm^2^)** | **PCSA:muscle**  **origin area index** |
| **ISP** | 54.25 | 467.82 | 0.116 |
| **SSP** | 75.15 | 403.40 | 0.186 |
| **ISP + SSP** | 130.84 | 871.22 | 0.150 |
| ***Massetognathus pascuali*** | | | |
|  | **Normalized**  **PCSA (mm^2^)** | **Normalized muscle**  **origin area (mm^2^)** | **PCSA:muscle**  **origin area index** |
| **ISP**  (*D. virginiana* ISP) | 29.40* | 253.49 | 0.116 |
| **ISP**  (*S. merianae* SPC) | 45.63 | 253.49 | 0.180 |
| **SSP**  (*D. virginiana* SSP) | 19.49 | 104.77 | 0.186 |
| **SSP**  (*S. merianae* SPC) | 18.86* | 104.77 | 0.180 |
| LOW ESTIMATE:  **ISP** (*D. virginiana* ISP)  **+ SSP** (*S. merianae* SPC) | 48.26* | 358.26 | 0.132 |
| HIGH ESTIMATE:  **ISP** (S*. merianae* SPC)  **+ SSP** (*D. virginiana* SSP) | 65.12 | 358.26 | 0.182 |
